# *N*-{3-[2-(4-Fluoro­phen­oxy)eth­yl]-2,4-dioxo-1,3-diaza­spiro­[4.5]decan-7-yl}-4-methyl­benzamide

**DOI:** 10.1107/S1600536811017946

**Published:** 2011-05-20

**Authors:** M. Vinduvahini, Binoy Krishna Saha, Mahalakhmi, H. D. Revanasiddappa, H. C. Devarajegowda

**Affiliations:** a Department of Physics, Sri D Devaraja Urs Govt. First Grade College, Hunsur 571 105, Mysore District, Karnataka, India; b Department of Chemistry, Pondicherry University, Pondicherry 605 014, India; c Department of Physics, Yuvaraja’s College (Constituent College), University of Mysore, Mysore 570 005, Karnataka, India; d Department of Studies in Chemistry, Manasagangotri, University of Mysore, Mysore 570 006, Karnataka, India

## Abstract

In the title compound, C_24_H_26_FN_3_O_4_, the two aromatic rings form a dihedral angle of 88.81 (15)°. The cyclo­hexane ring adopts a chair conformation and the five-membered ring is essentially planar, with a maximum deviation from planarity of 0.041 (2) Å. The crystal structure displays inter­molecular C—H⋯O and N—H⋯O hydrogen bonds.

## Related literature

For the biological activity of related compounds, see: Cartwright *et al.* (2007 ▶); Collins (2000 ▶); Warshakoon *et al.* (2006 ▶). For the pharmaceutical activity of related compounds, see: Kiselyov *et al.* (2006 ▶); Sakthivel & Cook (2005 ▶); Eldrup *et al.* (2004 ▶); Bamford *et al.* (2005 ▶); Puerstinger *et al.* (2006 ▶). For reference bond-length data, see: Allen *et al.* (1987 ▶).
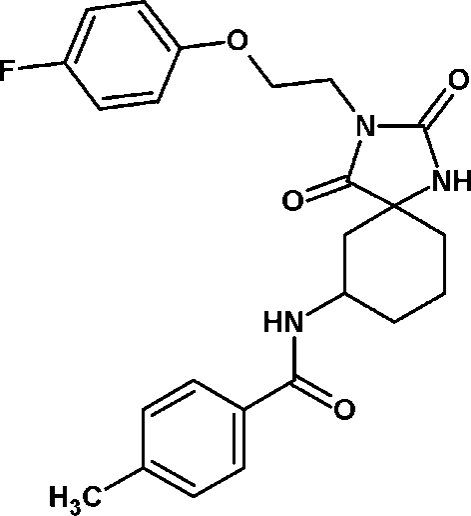


## Experimental

### 

#### Crystal data

C_24_H_26_FN_3_O_4_
*M* *_r_* = 439.48
Triclinic, 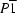

*a* = 9.1436 (17) Å
*b* = 10.103 (2) Å
*c* = 13.939 (2) Å
α = 99.239 (15)°
β = 106.550 (14)°
γ = 107.417 (18)°
*V* = 1134.5 (4) Å^3^
*Z* = 2
Mo *K*α radiation
μ = 0.09 mm^−1^
*T* = 293 K
0.22 × 0.15 × 0.12 mm

#### Data collection

Oxford Diffraction Xcalibur diffractometer
Absorption correction: multi-scan (*CrysAlis PRO RED*; Oxford Diffraction, 2010 ▶) *T*_min_ = 0.770, *T*_max_ = 1.000
7145 measured reflections
3967 independent reflections
2163 reflections with *I* > 2σ(*I*)
*R*_int_ = 0.045

#### Refinement

*R*[*F* ^2^ > 2σ(*F* ^2^)] = 0.052
*wR*(*F* ^2^) = 0.135
*S* = 0.90
3967 reflections
289 parameters
H-atom parameters constrained
Δρ_max_ = 0.21 e Å^−3^
Δρ_min_ = −0.19 e Å^−3^

### 

Data collection: *CrysAlis PRO CCD* (Oxford Diffraction, 2010 ▶); cell refinement: *CrysAlis PRO CCD*; data reduction: *CrysAlis PRO RED* (Oxford Diffraction, 2010 ▶); program(s) used to solve structure: *SHELXS97* (Sheldrick, 2008 ▶); program(s) used to refine structure: *SHELXL97* (Sheldrick, 2008 ▶); molecular graphics: *ORTEP-3* (Farrugia, 1997 ▶) and *CAMERON* (Watkin *et al.*, 1993 ▶); software used to prepare material for publication: *WinGX* (Farrugia, 1999 ▶).

## Supplementary Material

Crystal structure: contains datablocks I, global. DOI: 10.1107/S1600536811017946/wn2425sup1.cif

Structure factors: contains datablocks I. DOI: 10.1107/S1600536811017946/wn2425Isup2.hkl

Supplementary material file. DOI: 10.1107/S1600536811017946/wn2425Isup3.cml

Additional supplementary materials:  crystallographic information; 3D view; checkCIF report

## Figures and Tables

**Table 1:** Hydrogen-bond geometry (Å, °)

*D*—H⋯*A*	*D*—H	H⋯*A*	*D*⋯*A*	*D*—H⋯*A*
N7—H7⋯O5^i^	0.86	2.06	2.892 (3)	163
N8—H8⋯O4^ii^	0.86	2.22	3.060 (3)	165
C27—H27⋯O4^ii^	0.93	2.45	3.370 (3)	172

Symmetry codes: (i) 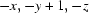; (ii) 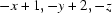.

**Fig. 1.**
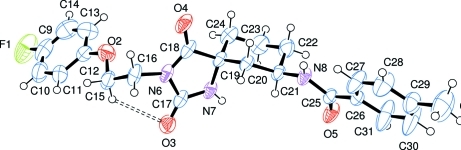
The title molecular structure with displacement ellipsoids drawn at the 50% probability level. The H atoms are shown as spheres of arbitrary radius.

**Fig. 2.**
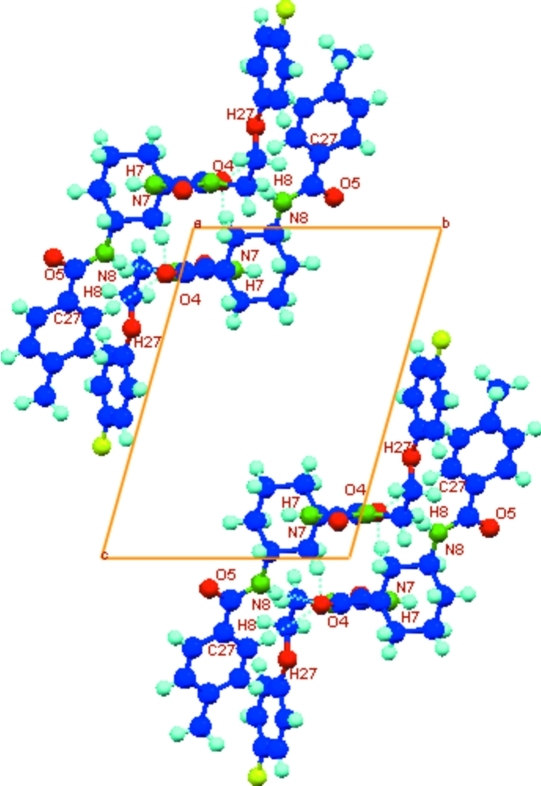
A view of the crystal structure, viewed down the a axis. Dashed lines indicate hydrogen bonds.

## supplementary crystallographic information

### Comment

One of the challenges of medicinal chemistry is the promotion of structural diversity, which can be achieved by the attachment of pharmacophoric groups to the significant molecular scaffold in combinatorial chemistry. Examples of such a process include *di* and *tri*-substituted hydantoins, which have been widely used in biological screenings, resulting in numerous pharmaceutical applications (Cartwright *et al.*, 2007; Collins, 2000; Warshakoon *et al.*, 2006). Hydantoin analogues have shown versatile therapeutic applications and some of them have been approved as drugs. For example, fosphenytoin as a sodium channel antagonist is used for the treatment of epilepsy. Phenytoin has antiarrhythmic, anticonvulsant, and antineuralgic activities. Ethotoin and mephenytoin both show anticonvulsant effects. Nilutamide is used in the treatment of prostate cancer (Kiselyov *et al.*, 2006; Sakthivel & Cook, 2005; Eldrup *et al.*, 2004; Bamford *et al.*, 2005; Puerstinger *et al.*, 2006).

The asymmetric unit of *N*-(3-(2-(4-fluorophenoxy)ethyl)-2,4- dioxo-1,3-diazaspiro[4.5]decan-7-yl)-4-methylbenzamide, C_24_H_26_FN_3_O_4_, contains just one molecule (Fig. 1). The two benzene rings (C9–C14) and (C26–C31) form a dihedral angle of 88.81 (15)°. The cyclohexane (C19–C24) ring adopts a chair conformation, and the five-membered ring is essentially planar, with a maximum deviation from planarity of 0.041 (2) Å for atom C17. Bond lengths (Allen *et al.*, 1987) and angles are normal.

The crystal structure displays intermolecular hydrogen bonds C27—H27···O4, N7—H7···O5 and N8—H8···O4 (Table 1 and Fig. 2). The packing of molecules in the crystal structure is depicted in Fig. 2.

### Experimental

*tert*-Butyl 4-oxocyclohexylcarbamate (5 g, 0.251 mol) and ammonium carbonate (4.99 g, 0.051 mol) were taken up in methanol (20 ml) and water (20 ml). A solution of sodium cyanide (2.41 g, 0.049 mol) in water (10 ml) was added dropwise and the reaction mixture stirred at RT for 24 hrs. It was then heated to 323 K for 2 days and cooled to RT. The resulting solid was filtered, washed with water and dried to yield hydantoin. This was taken up in acetonitrile (50 ml), K_2_CO_3_ (3.28 g, 0.023 mol) and 1-(2-bromoethoxy)-4- fluorobenzene (4.17 g, 0.019 mol) was added. The reaction mixture was heated at 358 K for 6 hrs, cooled to RT and filtered. The filtrate was concentrated to yield a white solid. The *tert*-butyl dicarbonate (BOC) was deprotected using dioxane-HCl (10 ml) and it was basified to obtain the free amine. The solid thus obtained was taken up (100 mg, 0.311 mmol) in dichloromethane (2 ml), and Et_3_N (0.2 ml) added. The mixture was then added to 4-methylbenzoyl chloride (57.7 mg, 0.373 mmol) and stirred at RT overnight. It was extracted in dichloromethane, concentrated, and purified using column chromatography over silica gel to yield the title compound (50 mg, 36.7%).

### Refinement

All H atoms were placed at calculated positions and refined using a riding model. N—H = 0.86 Å, C—H = 0.98 Å for methine, C—H = 0.97 Å for methylene, C—H = 0.93 Å for Csp^2^ and C—H = 0.96 Å for methyl. *U_iso_(H)* = 1.5*U_eq_(C)* for methyl H and 1.2*U_eq_(C, N)* for all other H atoms.

### Crystal data

**Table:**

C_24_H_26_FN_3_O_4_	*Z* = 2
*M**_r_* = 439.48	*F*(000) = 464
Triclinic, *P*1	*D*_x_ = 1.287 Mg m^−^^3^
Hall symbol: -P 1	Melting point: 419 K
*a* = 9.1436 (17) Å	Mo *K*α radiation, λ = 0.71073 Å
*b* = 10.103 (2) Å	Cell parameters from 3967 reflections
*c* = 13.939 (2) Å	θ = 2.7–25.0°
α = 99.239 (15)°	µ = 0.09 mm^−^^1^
β = 106.550 (14)°	*T* = 293 K
γ = 107.417 (18)°	Prism, colourless
*V* = 1134.5 (4) Å^3^	0.22 × 0.15 × 0.12 mm

### Data collection

**Table:**

Oxford Diffraction Xcalibur diffractometer	3967 independent reflections
Radiation source: fine-focus sealed tube	2163 reflections with *I* > 2σ(*I*)
graphite	*R*_int_ = 0.045
Detector resolution: 15.9821 pixels mm^-1^	θ_max_ = 25.0°, θ_min_ = 2.7°
ω scans	*h* = −10→10
Absorption correction: multi-scan (*CrysAlis PRO RED*; Oxford Diffraction, 2010)	*k* = −12→12
*T*_min_ = 0.770, *T*_max_ = 1.000	*l* = −16→16
7145 measured reflections	

### Refinement

**Table:**

Refinement on *F*^2^	Primary atom site location: structure-invariant direct methods
Least-squares matrix: full	Secondary atom site location: difference Fourier map
*R*[*F*^2^ > 2σ(*F*^2^)] = 0.052	Hydrogen site location: inferred from neighbouring sites
*wR*(*F*^2^) = 0.135	H-atom parameters constrained
*S* = 0.90	*w* = 1/[σ^2^(*F*_o_^2^) + (0.0758*P*)^2^] where *P* = (*F*_o_^2^ + 2*F*_c_^2^)/3
3967 reflections	(Δ/σ)_max_ < 0.001
289 parameters	Δρ_max_ = 0.21 e Å^−^^3^
0 restraints	Δρ_min_ = −0.19 e Å^−^^3^

### Special details

**Table:**

Experimental. *CrysAlis PRO*, Oxford Diffraction Ltd., Version 1.171.33.55 (release 05–01–2010 CrysAlis171. NET) Empirical absorption correction using spherical harmonics, implemented in SCALE3 ABSPACK scaling algorithm.^1^H NMR 400 MHz, DMSO-d_6_:δ 9.00 (s, 1H), 8.18 (d, J = 8.12 Hz, 1H), 7.72 (d, J = 8.16 Hz, 2H), 6.87–7.25 (m, 6H), 4.12 (q, J = 5.76 Hz, 3H), 3.71 (t, J = 5.84 Hz, 2H), 2.49–2.51 (m, 1H), 2.34 (s, 3H), 1.13–1.85 (m, 7H); MS:m/*z* 439.5 (*M*+), 440.5 (*M*+1); Anal.calcd for C_24_H_26_FN_3_O_4_: C, 65.59; H, 5.96; N, 9.56%; Found: C, 65.54; H, 5.92; N, 9.53%.
Geometry. All s.u.'s (except the s.u. in the dihedral angle between two l.s. planes) are estimated using the full covariance matrix. The cell s.u.'s are taken into account individually in the estimation of s.u.'s in distances, angles and torsion angles; correlations between s.u.'s in cell parameters are only used when they are defined by crystal symmetry. An approximate (isotropic) treatment of cell s.u.'s is used for estimating s.u.'s involving l.s. planes.
Refinement. Refinement of *F*^2^ against ALL reflections. The weighted *R*-factor *wR* and goodness of fit *S* are based on *F*^2^, conventional *R*-factors *R* are based on *F*, with *F* set to zero for negative *F*^2^. The threshold expression of *F*^2^ > 2σ(*F*^2^) is used only for calculating *R*-factors(gt) *etc*. and is not relevant to the choice of reflections for refinement. *R*-factors based on *F*^2^ are statistically about twice as large as those based on *F*, and *R*- factors based on ALL data will be even larger.

### Fractional atomic coordinates and isotropic or equivalent isotropic displacement parameters (Å^2^)

**Table:**

	*x*	*y*	*z*	*U*_iso_*/*U*_eq_	
F1	−0.5189 (2)	1.1248 (2)	−0.66068 (14)	0.1221 (8)	
O2	−0.0604 (2)	1.1356 (2)	−0.30513 (13)	0.0641 (5)	
O3	−0.1769 (2)	0.91403 (17)	−0.11175 (13)	0.0591 (5)	
O4	0.32910 (19)	1.06151 (17)	−0.14001 (13)	0.0621 (5)	
O5	0.18882 (18)	0.46876 (16)	0.09178 (12)	0.0594 (5)	
N6	0.0690 (2)	1.01919 (18)	−0.13490 (14)	0.0458 (5)	
N7	−0.0049 (2)	0.79320 (18)	−0.12864 (13)	0.0459 (5)	
H7	−0.0661	0.7084	−0.1313	0.055*	
N8	0.3607 (2)	0.67071 (18)	0.07755 (14)	0.0447 (5)	
H8	0.4547	0.7397	0.1050	0.054*	
C9	−0.4037 (4)	1.1332 (3)	−0.5707 (3)	0.0780 (10)	
C10	−0.4070 (4)	1.1951 (3)	−0.4784 (3)	0.0768 (9)	
H10	−0.4858	1.2350	−0.4762	0.092*	
C11	−0.2930 (3)	1.1989 (3)	−0.3869 (2)	0.0626 (7)	
H11	−0.2940	1.2426	−0.3231	0.075*	
C12	−0.1786 (3)	1.1383 (3)	−0.39054 (19)	0.0532 (7)	
C13	−0.1785 (3)	1.0763 (3)	−0.4859 (2)	0.0733 (8)	
H13	−0.1014	1.0347	−0.4891	0.088*	
C14	−0.2902 (4)	1.0748 (4)	−0.5763 (2)	0.0835 (10)	
H14	−0.2879	1.0343	−0.6404	0.100*	
C15	−0.0734 (3)	1.1746 (3)	−0.20599 (18)	0.0560 (7)	
H15A	−0.1757	1.1104	−0.2052	0.067*	
H15B	−0.0723	1.2721	−0.1916	0.067*	
C16	0.0680 (3)	1.1645 (2)	−0.12513 (19)	0.0585 (7)	
H16A	0.1691	1.2258	−0.1293	0.070*	
H16B	0.0656	1.2010	−0.0571	0.070*	
C17	−0.0530 (3)	0.9055 (2)	−0.12341 (17)	0.0447 (6)	
C18	0.2006 (3)	0.9841 (2)	−0.13550 (16)	0.0458 (6)	
C19	0.1618 (2)	0.8280 (2)	−0.12944 (16)	0.0393 (5)	
C20	0.2797 (3)	0.8245 (2)	−0.02788 (16)	0.0402 (6)	
H20A	0.3916	0.8675	−0.0249	0.048*	
H20B	0.2671	0.8808	0.0304	0.048*	
C21	0.2468 (3)	0.6706 (2)	−0.01947 (16)	0.0411 (6)	
H21	0.1354	0.6307	−0.0189	0.049*	
C22	0.2571 (3)	0.5788 (3)	−0.11345 (19)	0.0598 (7)	
H22A	0.3678	0.6149	−0.1138	0.072*	
H22B	0.2324	0.4805	−0.1083	0.072*	
C23	0.1382 (3)	0.5806 (3)	−0.21441 (19)	0.0645 (8)	
H23A	0.0267	0.5373	−0.2168	0.077*	
H23B	0.1504	0.5240	−0.2727	0.077*	
C24	0.1700 (3)	0.7340 (3)	−0.22326 (18)	0.0571 (7)	
H24A	0.0890	0.7334	−0.2860	0.069*	
H24B	0.2773	0.7738	−0.2282	0.069*	
C25	0.3252 (3)	0.5671 (2)	0.12644 (17)	0.0408 (6)	
C26	0.4538 (2)	0.5767 (2)	0.22404 (17)	0.0391 (5)	
C27	0.6002 (3)	0.6884 (3)	0.27164 (19)	0.0662 (8)	
H27	0.6238	0.7647	0.2420	0.079*	
C28	0.7148 (3)	0.6921 (3)	0.3625 (2)	0.0723 (9)	
H28	0.8133	0.7705	0.3921	0.087*	
C29	0.6874 (3)	0.5846 (3)	0.40928 (19)	0.0638 (8)	
C30	0.5429 (4)	0.4727 (4)	0.3620 (3)	0.1180 (16)	
H30	0.5198	0.3969	0.3922	0.142*	
C31	0.4285 (3)	0.4671 (3)	0.2704 (3)	0.1025 (13)	
H31	0.3321	0.3868	0.2397	0.123*	
C32	0.8132 (4)	0.5897 (4)	0.5090 (2)	0.1124 (14)	
H32A	0.7719	0.5052	0.5309	0.169*	
H32B	0.8347	0.6739	0.5619	0.169*	
H32C	0.9127	0.5933	0.4975	0.169*	

### Atomic displacement parameters (Å^2^)

**Table:**

	*U*^11^	*U*^22^	*U*^33^	*U*^12^	*U*^13^	*U*^23^
F1	0.1175 (15)	0.1150 (16)	0.0906 (13)	0.0165 (13)	−0.0120 (12)	0.0524 (12)
O2	0.0677 (11)	0.0840 (14)	0.0567 (11)	0.0419 (10)	0.0252 (9)	0.0281 (10)
O3	0.0558 (11)	0.0495 (10)	0.0745 (12)	0.0177 (9)	0.0234 (10)	0.0255 (9)
O4	0.0522 (10)	0.0471 (10)	0.0820 (12)	0.0029 (8)	0.0205 (9)	0.0392 (9)
O5	0.0469 (10)	0.0457 (10)	0.0702 (11)	−0.0024 (8)	0.0085 (9)	0.0339 (9)
N6	0.0492 (11)	0.0292 (10)	0.0504 (11)	0.0061 (9)	0.0094 (10)	0.0186 (9)
N7	0.0410 (10)	0.0278 (10)	0.0575 (12)	0.0006 (8)	0.0088 (9)	0.0200 (9)
N8	0.0381 (10)	0.0322 (10)	0.0571 (11)	0.0031 (8)	0.0111 (9)	0.0236 (9)
C9	0.078 (2)	0.071 (2)	0.067 (2)	0.0094 (17)	0.0062 (18)	0.0400 (18)
C10	0.077 (2)	0.075 (2)	0.091 (2)	0.0356 (17)	0.0264 (19)	0.0465 (19)
C11	0.0764 (19)	0.0647 (18)	0.0670 (17)	0.0395 (16)	0.0321 (16)	0.0322 (15)
C12	0.0574 (15)	0.0552 (15)	0.0560 (16)	0.0213 (13)	0.0253 (14)	0.0277 (13)
C13	0.0655 (18)	0.088 (2)	0.067 (2)	0.0270 (17)	0.0276 (16)	0.0163 (17)
C14	0.094 (2)	0.084 (2)	0.0567 (19)	0.011 (2)	0.0255 (19)	0.0180 (17)
C15	0.0770 (18)	0.0440 (14)	0.0574 (16)	0.0267 (13)	0.0279 (14)	0.0244 (13)
C16	0.0807 (18)	0.0293 (12)	0.0588 (15)	0.0152 (12)	0.0164 (14)	0.0185 (12)
C17	0.0451 (14)	0.0358 (13)	0.0418 (13)	0.0047 (11)	0.0057 (11)	0.0175 (11)
C18	0.0488 (14)	0.0351 (12)	0.0381 (12)	0.0004 (12)	0.0041 (11)	0.0182 (11)
C19	0.0408 (12)	0.0308 (11)	0.0415 (13)	0.0054 (10)	0.0116 (10)	0.0168 (10)
C20	0.0437 (12)	0.0306 (12)	0.0428 (12)	0.0062 (10)	0.0149 (11)	0.0155 (10)
C21	0.0406 (12)	0.0327 (12)	0.0490 (13)	0.0083 (10)	0.0150 (11)	0.0196 (11)
C22	0.0767 (18)	0.0399 (14)	0.0686 (17)	0.0234 (13)	0.0290 (15)	0.0192 (13)
C23	0.089 (2)	0.0459 (15)	0.0531 (15)	0.0195 (14)	0.0259 (15)	0.0058 (13)
C24	0.0681 (17)	0.0527 (15)	0.0434 (14)	0.0113 (13)	0.0174 (13)	0.0190 (13)
C25	0.0421 (12)	0.0301 (12)	0.0529 (13)	0.0106 (10)	0.0192 (11)	0.0191 (11)
C26	0.0404 (12)	0.0315 (12)	0.0490 (13)	0.0121 (10)	0.0186 (11)	0.0175 (10)
C27	0.0681 (17)	0.0468 (15)	0.0579 (15)	−0.0049 (13)	0.0059 (14)	0.0257 (13)
C28	0.0620 (17)	0.0583 (18)	0.0632 (17)	−0.0048 (14)	−0.0006 (14)	0.0215 (15)
C29	0.0542 (15)	0.0684 (18)	0.0593 (16)	0.0170 (14)	0.0058 (13)	0.0281 (14)
C30	0.078 (2)	0.092 (2)	0.133 (3)	−0.0113 (19)	−0.023 (2)	0.086 (2)
C31	0.0685 (19)	0.070 (2)	0.117 (3)	−0.0167 (16)	−0.0207 (18)	0.067 (2)
C32	0.082 (2)	0.121 (3)	0.092 (2)	0.010 (2)	−0.0166 (19)	0.055 (2)

### Geometric parameters (Å, °)

**Table:**

F1—C9	1.360 (3)	C19—C24	1.522 (3)
O2—C12	1.372 (3)	C19—C20	1.529 (3)
O2—C15	1.423 (3)	C20—C21	1.523 (3)
O3—C17	1.216 (3)	C20—H20A	0.9700
O4—C18	1.226 (2)	C20—H20B	0.9700
O5—C25	1.239 (2)	C21—C22	1.522 (3)
N6—C18	1.355 (3)	C21—H21	0.9800
N6—C17	1.408 (3)	C22—C23	1.520 (3)
N6—C16	1.455 (3)	C22—H22A	0.9700
N7—C17	1.332 (3)	C22—H22B	0.9700
N7—C19	1.462 (3)	C23—C24	1.521 (3)
N7—H7	0.8600	C23—H23A	0.9700
N8—C25	1.348 (2)	C23—H23B	0.9700
N8—C21	1.453 (3)	C24—H24A	0.9700
N8—H8	0.8600	C24—H24B	0.9700
C9—C10	1.350 (4)	C25—C26	1.490 (3)
C9—C14	1.353 (4)	C26—C27	1.362 (3)
C10—C11	1.385 (4)	C26—C31	1.364 (3)
C10—H10	0.9300	C27—C28	1.381 (3)
C11—C12	1.369 (3)	C27—H27	0.9300
C11—H11	0.9300	C28—C29	1.351 (3)
C12—C13	1.377 (4)	C28—H28	0.9300
C13—C14	1.372 (4)	C29—C30	1.352 (4)
C13—H13	0.9300	C29—C32	1.513 (4)
C14—H14	0.9300	C30—C31	1.381 (4)
C15—C16	1.496 (3)	C30—H30	0.9300
C15—H15A	0.9700	C31—H31	0.9300
C15—H15B	0.9700	C32—H32A	0.9600
C16—H16A	0.9700	C32—H32B	0.9600
C16—H16B	0.9700	C32—H32C	0.9600
C18—C19	1.531 (3)		
			
C12—O2—C15	118.2 (2)	C21—C20—H20B	109.4
C18—N6—C17	111.22 (18)	C19—C20—H20B	109.4
C18—N6—C16	123.88 (18)	H20A—C20—H20B	108.0
C17—N6—C16	123.7 (2)	N8—C21—C22	112.08 (19)
C17—N7—C19	113.54 (17)	N8—C21—C20	109.75 (17)
C17—N7—H7	123.2	C22—C21—C20	110.07 (16)
C19—N7—H7	123.2	N8—C21—H21	108.3
C25—N8—C21	122.74 (17)	C22—C21—H21	108.3
C25—N8—H8	118.6	C20—C21—H21	108.3
C21—N8—H8	118.6	C23—C22—C21	111.5 (2)
C10—C9—C14	121.4 (3)	C23—C22—H22A	109.3
C10—C9—F1	120.1 (4)	C21—C22—H22A	109.3
C14—C9—F1	118.5 (4)	C23—C22—H22B	109.3
C9—C10—C11	119.9 (3)	C21—C22—H22B	109.3
C9—C10—H10	120.0	H22A—C22—H22B	108.0
C11—C10—H10	120.0	C22—C23—C24	110.7 (2)
C12—C11—C10	119.8 (3)	C22—C23—H23A	109.5
C12—C11—H11	120.1	C24—C23—H23A	109.5
C10—C11—H11	120.1	C22—C23—H23B	109.5
C11—C12—O2	124.8 (2)	C24—C23—H23B	109.5
C11—C12—C13	118.9 (3)	H23A—C23—H23B	108.1
O2—C12—C13	116.3 (3)	C23—C24—C19	110.85 (17)
C14—C13—C12	121.1 (3)	C23—C24—H24A	109.5
C14—C13—H13	119.5	C19—C24—H24A	109.5
C12—C13—H13	119.5	C23—C24—H24B	109.5
C9—C14—C13	118.9 (3)	C19—C24—H24B	109.5
C9—C14—H14	120.5	H24A—C24—H24B	108.1
C13—C14—H14	120.5	O5—C25—N8	120.7 (2)
O2—C15—C16	108.8 (2)	O5—C25—C26	121.51 (17)
O2—C15—H15A	109.9	N8—C25—C26	117.78 (18)
C16—C15—H15A	109.9	C27—C26—C31	115.8 (2)
O2—C15—H15B	109.9	C27—C26—C25	124.64 (18)
C16—C15—H15B	109.9	C31—C26—C25	119.5 (2)
H15A—C15—H15B	108.3	C26—C27—C28	122.2 (2)
N6—C16—C15	114.0 (2)	C26—C27—H27	118.9
N6—C16—H16A	108.7	C28—C27—H27	118.9
C15—C16—H16A	108.7	C29—C28—C27	121.6 (2)
N6—C16—H16B	108.7	C29—C28—H28	119.2
C15—C16—H16B	108.7	C27—C28—H28	119.2
H16A—C16—H16B	107.6	C28—C29—C30	116.6 (2)
O3—C17—N7	128.92 (19)	C28—C29—C32	121.3 (2)
O3—C17—N6	124.1 (2)	C30—C29—C32	122.1 (2)
N7—C17—N6	106.9 (2)	C29—C30—C31	122.2 (2)
O4—C18—N6	127.1 (2)	C29—C30—H30	118.9
O4—C18—C19	125.1 (2)	C31—C30—H30	118.9
N6—C18—C19	107.82 (17)	C26—C31—C30	121.5 (2)
N7—C19—C24	112.72 (18)	C26—C31—H31	119.2
N7—C19—C20	111.68 (16)	C30—C31—H31	119.2
C24—C19—C20	111.41 (19)	C29—C32—H32A	109.5
N7—C19—C18	99.91 (19)	C29—C32—H32B	109.5
C24—C19—C18	111.22 (16)	H32A—C32—H32B	109.5
C20—C19—C18	109.34 (17)	C29—C32—H32C	109.5
C21—C20—C19	111.03 (17)	H32A—C32—H32C	109.5
C21—C20—H20A	109.4	H32B—C32—H32C	109.5
C19—C20—H20A	109.4		
			
C14—C9—C10—C11	−0.4 (4)	O4—C18—C19—C20	64.1 (3)
F1—C9—C10—C11	177.8 (2)	N6—C18—C19—C20	−115.9 (2)
C9—C10—C11—C12	−0.8 (4)	N7—C19—C20—C21	71.2 (2)
C10—C11—C12—O2	−179.9 (2)	C24—C19—C20—C21	−55.8 (2)
C10—C11—C12—C13	0.9 (4)	C18—C19—C20—C21	−179.18 (19)
C15—O2—C12—C11	11.8 (3)	C25—N8—C21—C22	−84.7 (2)
C15—O2—C12—C13	−168.9 (2)	C25—N8—C21—C20	152.6 (2)
C11—C12—C13—C14	0.1 (4)	C19—C20—C21—N8	179.89 (18)
O2—C12—C13—C14	−179.2 (2)	C19—C20—C21—C22	56.1 (2)
C10—C9—C14—C13	1.4 (4)	N8—C21—C22—C23	−179.60 (16)
F1—C9—C14—C13	−176.8 (2)	C20—C21—C22—C23	−57.2 (2)
C12—C13—C14—C9	−1.2 (4)	C21—C22—C23—C24	57.2 (3)
C12—O2—C15—C16	−179.74 (19)	C22—C23—C24—C19	−55.8 (3)
C18—N6—C16—C15	129.5 (2)	N7—C19—C24—C23	−71.1 (2)
C17—N6—C16—C15	−64.1 (3)	C20—C19—C24—C23	55.4 (3)
O2—C15—C16—N6	−64.6 (2)	C18—C19—C24—C23	177.7 (2)
C19—N7—C17—O3	−172.4 (2)	C21—N8—C25—O5	−2.8 (3)
C19—N7—C17—N6	8.0 (2)	C21—N8—C25—C26	178.4 (2)
C18—N6—C17—O3	173.5 (2)	O5—C25—C26—C27	−173.7 (2)
C16—N6—C17—O3	5.6 (3)	N8—C25—C26—C27	5.1 (3)
C18—N6—C17—N7	−6.9 (2)	O5—C25—C26—C31	7.3 (4)
C16—N6—C17—N7	−174.82 (19)	N8—C25—C26—C31	−173.8 (3)
C17—N6—C18—O4	−176.8 (2)	C31—C26—C27—C28	−1.3 (4)
C16—N6—C18—O4	−8.9 (4)	C25—C26—C27—C28	179.7 (3)
C17—N6—C18—C19	3.2 (2)	C26—C27—C28—C29	−0.2 (5)
C16—N6—C18—C19	171.07 (18)	C27—C28—C29—C30	0.7 (5)
C17—N7—C19—C24	−124.0 (2)	C27—C28—C29—C32	−179.7 (3)
C17—N7—C19—C20	109.7 (2)	C28—C29—C30—C31	0.2 (6)
C17—N7—C19—C18	−5.9 (2)	C32—C29—C30—C31	−179.4 (4)
O4—C18—C19—N7	−178.6 (2)	C27—C26—C31—C30	2.2 (5)
N6—C18—C19—N7	1.4 (2)	C25—C26—C31—C30	−178.7 (3)
O4—C18—C19—C24	−59.4 (3)	C29—C30—C31—C26	−1.7 (6)
N6—C18—C19—C24	120.6 (2)		

### Hydrogen-bond geometry (Å, °)

**Table:**

*D*—H···*A*	*D*—H	H···*A*	*D*···*A*	*D*—H···*A*
N7—H7···O5^i^	0.86	2.06	2.892 (3)	163
N8—H8···O4^ii^	0.86	2.22	3.060 (3)	165
C27—H27···O4^ii^	0.93	2.45	3.370 (3)	172

Symmetry codes: (i) −*x*, −*y*+1, −*z*; (ii) −*x*+1, −*y*+2, −*z*.
